# Development of a 17-Plex of Penta- and Tetra-Nucleotide Microsatellites for DNA Profiling and Paternity Testing in Horses

**DOI:** 10.3389/fvets.2022.861623

**Published:** 2022-04-07

**Authors:** Andrea M. Luttman, Misa Komine, Tuddow Thaiwong, Tyler Carpenter, Susan L. Ewart, Matti Kiupel, Ingeborg M. Langohr, Patrick J. Venta

**Affiliations:** ^1^Microbiology and Molecular Genetics, College of Veterinary Medicine, Michigan State University, East Lansing, MI, United States; ^2^Genetics and Genomic Sciences, Michigan State University, East Lansing, MI, United States; ^3^Pathobiology and Diagnostic Investigation, College of Veterinary Medicine, Michigan State University, East Lansing, MI, United States; ^4^Veterinary Diagnostic Laboratory, College of Veterinary Medicine, Michigan State University, East Lansing, MI, United States; ^5^Department of Obstetrics, Gynecology and Reproductive Biology, Michigan State University College of Human Medicine, Grand Rapids, MI, United States; ^6^Large Animal Clinical Sciences, College of Veterinary Medicine, Michigan State University, East Lansing, MI, United States; ^7^Pathobiological Sciences, School of Veterinary Medicine, Louisiana State University, Baton Rouge, LA, United States; ^8^Small Animal Clinical Sciences, College of Veterinary Medicine, Michigan State University, East Lansing, MI, United States

**Keywords:** tetranucleotide short tandem repeat, horse, 17-plex, DNA profiling, Friesian, paternity testing, equine

## Abstract

Tetranucleotide and pentanucleotide short tandem repeat (hereafter termed tetraSTR and pentaSTR) polymorphisms have properties that make them desirable for DNA profiling and paternity testing. However, certain species, such as the horse, have far fewer tetraSTRs than other species and for this reason dinucleotide STRs (diSTRs) have become the standard for DNA profiling in horses, despite being less desirable for technical reasons. During our testing of a series of candidate genes as potentially underlying a heritable condition characterized by megaesophagus in the Friesian horse breed, we found that good tetraSTRs do exist in horses but, as expected, at a much lower frequency than in other species, e.g., dogs and humans. Using a series of efficient methods developed in our laboratory for the production of multiplexed tetraSTRs in other species, we identified a set of tetra- and pentaSTRs that we developed into a 17-plex panel for the horse, plus a sex-identifying marker near the amelogenin gene. These markers were tested in 128 horses representing 16 breeds as well as crossbred horses, and we found that these markers have useful genetic variability. Average observed heterozygosities (Ho) ranged from 0.53 to 0.89 for the individual markers (0.66 average Ho for all markers), and 0.62-0.82 for expected heterozygosity (He) within breeds (0.72 average He for all markers). The probability of identity (PI) within breeds for which 10 or more samples were available was at least 1.1 x 10^−11^, and the PI among siblings (PIsib) was 1.5 x 10^−5^. Stutter was ≤ 11% (average stutter for all markers combined was 6.9%) compared to the more than 30% typically seen with diSTRs. We predict that it will be possible to develop accurate allelic ladders for this multiplex panel that will make cross-laboratory comparisons easier and will also improve DNA profiling accuracy. Although we were only able to exclude candidate genes for Friesian horse megaesophagus with no unexcluded genes that are possibly causative at this point in time, the study helped us to refine the methods used to develop better tetraSTR multiplexed panels for species such as the horse that have a low frequency of tetraSTRs.

## Introduction

Horses have been used by humans for centuries for help in farming, transportation, mounting soldiers, companionship, and other uses. Because of the long-term association of people with horses, horses have remained an important part of our civilization. According to the American Horse Council, horses contribute over $50 billion dollars in direct economic impact to the United States economy via an estimated 7.2 million horses (https://www.horsecouncil.org/resources/economics/). In order to keep track of the ownership of these horses and to maintain accurate pedigrees, dinucleotide short tandem repeat (diSTR) polymorphism have been utilized as a means of DNA identification. While tetranucleotide short tandem repeat (tetraSTR) polymorphisms have been used in dogs and humans for DNA profiling and paternity testing, the much lower number of tetraSTRs in horses has so far precluded similar testing in horses (1, 2). During our work to test a number of candidate genes that could underlie the heritable condition characterized by megaesophagus in the Friesian horse breed, we discovered information that led us to develop a 17-plex panel of tetranucleotide and pentanucleotide markers (3, 4).

Friesian horse megaesophagus is perceived to be a heritable disorder in the breed (3). It causes a variety of esophageal lesions that often lead to the horse's death. If the presumed gene variant underlying this disorder could be found, it would be possible to develop a diagnostic test for carriers of the mutation, and a way for Friesian horse breeders to conduct their breeding programs to eliminate this condition. As a first attempt to identify the underlying causative gene, we used an approach we call “exclusion analysis” that is inexpensive and can generally result in either excluding all candidate genes in a moderately inbred domestic animal population or, on occasion, result in a lack of exclusion that may lead to the identification of a causative gene, such as the one (*SLC45A2*) that underlies white coat color in Doberman pinschers (5, 6).

There is a common perception that tetraSTRs with good heterozygosity (which we define for this report as those above 0.50 expected heterozygosity) are quite rare in the horse genome, even though in other species, such as dogs, there is an abundance of them (7, 8). For this reason, microsatellites developed for use in horses have been predominantly diSTRs [e.g., (9–11)]. However, while diSTRs have been useful and have some advantages as markers for DNA profiling (e.g., smaller amplicon sizes and a greater abundance in virtually all eukaryotes), they also have several disadvantages compared to longer motif microsatellites such as tetraSTRs (1, 12). Stutter bands, which are peaks that show up as one or more whole repeats less than the true allele as a result of strand slippage during PCR DNA synthesis, are a more significant problem for shorter repeat unit sizes such as diSTRs, for which stutter is often above 30%, thereby making automated scoring problematic (13). In addition, diSTR multi-step mutations are not unusual (>30% of mutations observed in humans), making it more difficult to account for these events when doing parentage analysis (14). It has also been difficult to develop and implement useful diSTR marker ladders, which are commonly used with tetraSTRs such as those used in human parentage analysis (1). The lack of such ladders also makes inter-laboratory comparisons for called alleles more difficult (15). Although SNP-based markers are under development to replace diSTRs for DNA profiling of horses, they have currently not been implemented on a widespread basis. Furthermore, prior to implementation, problems have only been partly overcome, which include determining the amount of heterozygosity of the chosen markers (within and among horse breeds), the number of markers needed to achieve useful values, potential problems of linkage disequilibrium, and the current lack of instrumentation among service-providing laboratories [e.g., (16, 17)].

As we developed our markers for testing several candidate genes for the Friesian horse condition, it became obvious that, while good tetraSTRs exist only in relatively low numbers in the horse, there were sufficient numbers to develop a multiplex panel for equine DNA profiling. Here we report upon the methods used to develop the panel as well as the values for the important genetic parameters for the panel itself.

## Materials and Methods

### DNA Samples

DNA samples were originally isolated from blood by a sodium perchlorate extraction technique (18). These samples were obtained from remaining aliquots of previous studies (19). Samples from the following breeds (with numbers of samples) were used during the development of the multiplex panel: Quarter horse (18); Arabian (16); Thoroughbred (13); Paint (11); Standardbred (10); Miniature horse (6); Morgan (6); Haflinger (4); Percheron (4); Pinto (4); American Saddlebred (4); Appaloosa (3); Hanoverian (3); Friesian (2); Tennessee Walker (2); Belgian (1); Anglo-Arab (1); Morab (1); American Paso Fino (1); Paso Fino (1) and mixed breed horses (12). Additional samples from affected and unaffected Friesian horses (not counting the two Friesians listed above) were collected from blood samples or tissues from horses that died after developing megaesophagus. Lastly, some additional samples were collected from hair roots from an Arabian horse breeding farm to determine usability of this DNA source. DNA was isolated from 20 hair roots using QIAGEN DNeasy Blood and Tissue kit as per the manufacturer's instructions. DNA samples from several other species (mouse, cat, dog, cow, and goat) were generously supplied by other labs on campus. DNA samples from these other species were examined, as is commonly done with microsatellites that might be used for forensic purposes. The rule for examining other species is based on the human forensics Scientific Working Group on DNA Analysis Methods (SWGDAM) validation guideline 3.2 “species specificity” (https://www.swgdam.org/publications). The goal is to prevent misinterpretation of misidentified or mixed DNA samples. Although this guideline has not been a requirement in animal forensic testing, it is nevertheless often followed during the validation process. DNA samples were collected under IACUC approval.

### Identification of Useful TetraSTRs by the SW Score Method

We had previously noted that tetraSTRs with Smith-Waterman (SW) scores > 450 for dogs and humans (UCSC Genome Browser prior to 2012) had useful heterozygosity (>0.80 of the maximum expected heterozygosity in both humans and purebred dogs; Venta, unpublished results). We used the same strategy to identify tetra- and pentaSTRs in the horse genome using the UCSC Genome Browser's table browser function. In later stages of the work, a second critical feature of the STRs was identified with respect to heterozygosity. The longest uninterrupted sequence (LUS) had to be eight or greater for the SW score to adequately predict the heterozygosity for a given population. In order to produce useful primer sets as conveniently as possible, STRs were chosen that also had a few hundred bp of unique flanking DNA (i.e., areas that do not contain repetitive elements such as SINEs or LINEs) that was less likely to produce off-target amplicons. Amplicons that would include other STRs (e.g., diSTRs and monoSTRs) that would confound the interpretation of the tetraSTR were also avoided. In addition, only one STR was chosen per horse chromosome, except for chromosome 4 for which two STRs were chosen that are 68 Mb apart.

### Design of Primers

When potentially useful tetra- and pentaSTRs were identified near the candidate genes (generally within 2 Mb), the sequences plus several hundred bp of flanking sequence were searched for PCR primer pairs using Primer3 software (http://bioinfo.ut.ee/primer3-0.4.0/). Many primers were chosen for which the 3′ end finished in two adenines (AA) for reasons that will be discussed later. As primer sets accumulated, additional effort was put into identifying sets that would not encompass overlapping ranges with other markers labeled with the same fluorescent dye. When marker ranges were discovered to overlap, new primers were designed to adjust the size range for one of the markers that showed the overlap. Forward (F) primers were labeled with either 6-FAM (hereafter referred to as FAM), or HEX, both from IDT (idtdna.com, USA) or NED (from ABI, USA, obtained through Thermofisher.com, USA). Unlabeled primers were purchased from IDT. Primer sequences used in the final multiplex panel are listed in Table 1 and other primers are listed in Supplementary Tables S1, S2. Primers were initially tested for robust amplification and good heterozygosity as single amplicons using a universal primer labeling system (see below in section Universal and directly labeled primers). Many reverse primers included an additional pigtail or a single “g” to suppress split peaks (20, 23). Occasionally (e.g., for the amelogenin marker) extra bases were added to avoid overlap with other markers or pull-up peaks. Primers were also checked using Autodimer for interactions that might occur during multiplex analysis [(24); https://www-s.nist.gov/dnaAnalysis/]. Our primary criteria were that no pairwise primer interactions were found that had a score of seven or more (AutoDimer output), and that the reverse primer did not template more than two bases at the 3′ end within the forward primer. Some care was also taken to keep the LUS shorter than 16 to reduce the problem of large stutter bands (25).

**Table 1:** Marker primers, observed data, and mutation rate predictions.

**Marker**	**Primer sequences**	**Motif (EquCab2)**	**SWS^a^**	**Label**	**conc. (μM)**	**Ref size**	**range**	**wt avg^b^**	**alleles**	**stutter**	**mut rate**
amel(YX)	F: CCAGGATGAGGTGGTAGCTTTTATA	[GA]12	NA	HEX	0.7	113;134	113-150	134	11	ND	NA
	R: gtATGTGAACAATTGCATATTGACTTAATCT				0.7						
ECA28.003.6	F: aGGTAGCATAACCCCTACTGAGATAA	[AGAT]13	468	HEX	0.3	179	159-187	171	8	10.3	0.18
	R: gtttcttGGGTTCCACATGTCAAAACAAA				0.3						
ECA15.001.6	F: TGCTTGGTGTACAGGCCTCAG	[TCTA]4[TCTG][TCTA]11	602	HEX	1.3	245	228-257	242	8	9.1	0.33
	R: gtttcttTTTCTGAGAGAAAGCTGAAAGTATG				1.3						
ECA07.065.4	F: aGAACAATGAGCAGGGAGTACAA	[ATAG]13	477	HEX	1.0	291	267-305	283	8	7.9	0.19
	R: gAGCAAGACTTGAAGAGGAATGGA				1.0						
ECA05.065.8	F: GGACTTTCAAAACTCACCCAAA	[GTTTT]11	504	HEX	1.5	347	312-361	336	8	1.7	0.22
	R: GATACAAAGTCCATGATCAAAACAAA				1.5						
ECA12.004.8	F: AAGGAGCAAGTTCAGGCAAA	[ATCT]*ATC* [ATCT]2[ATCC] [ATCT][ATTG]TCT[ATCT]9 **ATC**[ATCT]3[ATCC]2[ATCT]	633	HEX	5.0	431	410-435	420	9	3.2	0.37
	R: gtttcttTCATCCCTTGTACGCCTCTAA				5.0						
ECA24.028.9	F: TTTCAGGTTCTCGTTACTCAGATAGAA	[TCTCT]11	495	NED	1.0	120	80-137	113	9	6.2	0.21
	R: ATATTCTTGTAGGTAGGGTTT				1.0						
ECA22.012.1	F: CTCATGGTCTTTTAATTTTGAGTTATAC	[AGAT]12	510	NED	0.5	169	142-172	160	7	2.5	0.23
	R: gtttcttGCAACACATGTAACTGACCCAAA				0.5						
ECA21.027.5	F: TCCAAGGACCTTCTTCCAAA	[GATA]2[GTTA][GATA] [GACA][GATA]2 *GAT*[GATA]11 [GATC]	608	NED	1.5	221	193-236	215	6	7.3	0.34
	R: gtttcttAAATTAGTGAATTTGGAGAAAACAA				1.5						
ECA25.011.9	F: TCTGAGAGGTGATGGCAAAA	[ATCT]3[ATCC][ATCT]11 [ATCC]4	602	NED	1.0	248	235-267	250	8	10.7	0.33
	R: gtttcttTTCATTGTGTACAGTGTGGTATCAA				1.0						
ECA27.020.1	F: TTGAATTGCCATGATTAGGAA	[AGAT]12	582	NED	2.5	307	285-326	306	7	7.3	0.31
	R: gTGAATTTGGGCTGAGATTGAA				2.5						
ECA14.076.8	F: CCTACTTAGTCCCCCTTCCTGAAA	[CATC]2[TATC][CATT] [TATC][CATC]4[CAAC] [CATC][CAAC][CATC]10	663	NED	1.5	354	346-362	356	4	8.0	0.42
	R: gtttcttCAGGACAGAGGTTAAGTCACAAATAA				1.5						
ECA04.104.2	F: AGGAGTGGCAGTTGGTTGTGG	[CTAT] *GTG* [CTAT]9 *CAT* [CTAT][CTGT][CTAA] *T* [CTAT]CAT[CTAT]4[CTAG] T[CTAT] *CAT* [CTAT]2	931	NED	2.5	428	411-436	422	7	6.6	0.92
	R: gtttcttCACCCATCAATGCACAAATCTGCAGAAAA				2.5						
ECA04.035.5	F: CCCACATGACAAAAGCACAA	[TAAAA]9	405	6-FAM	0.4	127	97-131	114	7	1.2	0.13
	R: gTTCTCTCTGAGTCCAGATGCAA				0.4						
ECA02.105.5	F: AGCTCTTGAGCCCTCTTTGTAA	[AGAA] *CT* [AGAA]14 [AGGA]*A*[AGAA]2 [GGAA]2[AGAT]2	647	6-FAM	1.0	198	172-207	191	12	6.6	0.39
	R: gtttcttTTCCTTCATGCTGTTCCTGTAA				1.0						
ECA03.025.9	F: AGTAACATTTGGGTCATCTGAAA	[AGAT]5 *GAT* [AGGT] [AGAT]10	542	6-FAM	1.0	254	238-261	252	7	7.5	0.26
	R: gCGCAGCTCCTCATACTGAAA				1.0						
ECA01.102.4	F: TTTGGAGATGTTGGAAGTTAAGG	[ATCT]7 *ATC* [ATCT]12	671	6-FAM	1.0	296	261-307	297	9	9.8	0.43
	R: gtttcttAAGGGGAGAGGATGGAGCAAGAAAGAA				1.0						
ECA20.003.8	F: AAATAAGATGAATAGACAGGCCCTAA	[TATC]18[TGTC]	672	6-FAM	4.8	409	354-417	381	17	11.2	0.43
	R: gtttcttTCCGACTCATCCTACAGCAA				4.8						

*The table is sorted by primer label (HEX, NED, and 6-FAM) and then by the amplicon size predicted from the reference genome (Ref size)*.

*Markers are named by the chromosome upon which they reside (Eca, Equus caballus), followed by the megabase position for the repeat from the horse 2007 (EquCAb2) build*.

*Lower case letters within the primer sequences indicate nucleotides that are not found in the horse reference genome; these nucleotides are primarily “pigtails” to suppress split peaks (20)*.

**Figure 1:**
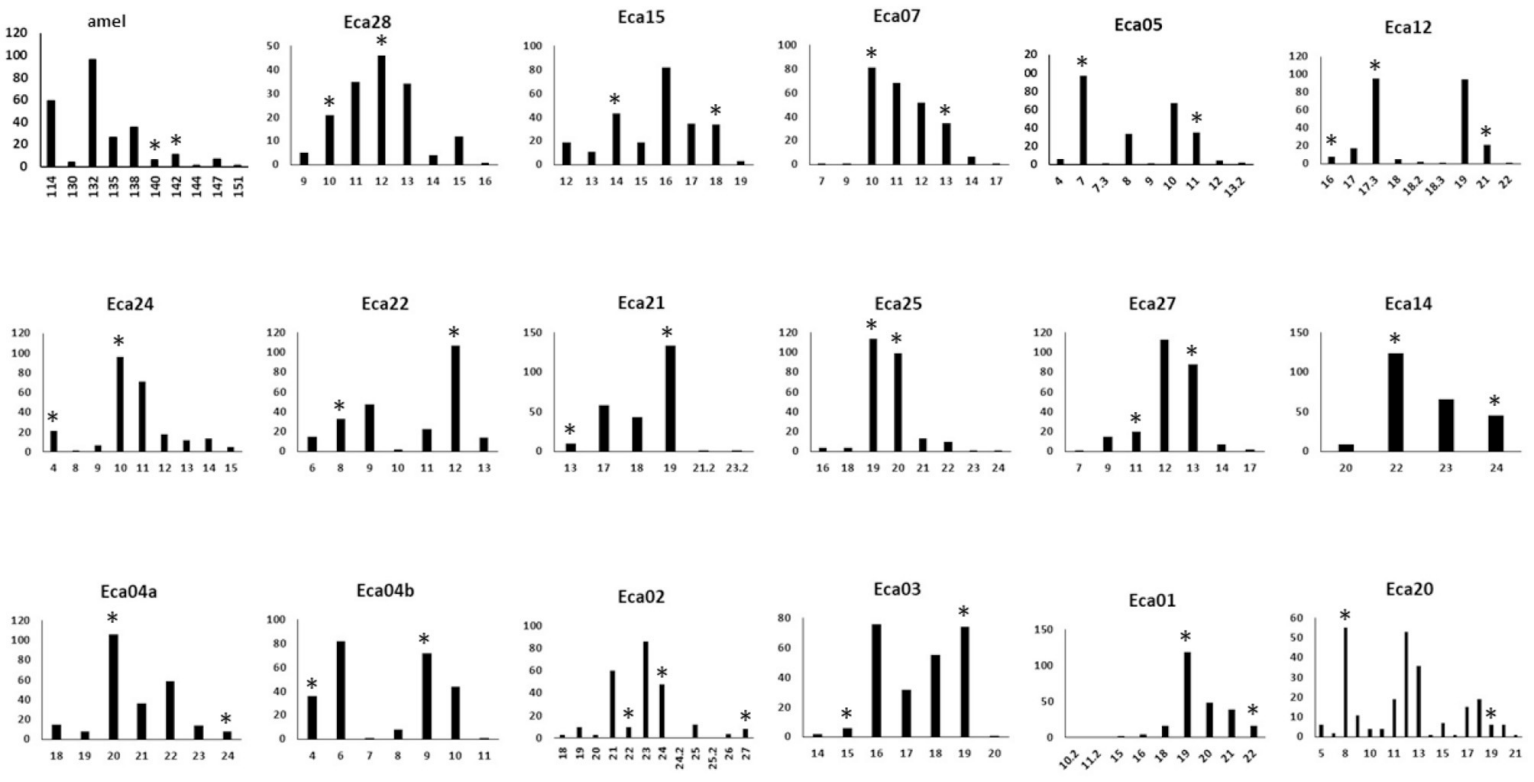
Histograms of allele frequencies for markers across 124 horses with the 17-plex panel plus the amel marker. Allele numbers are on the X-axis and the allele count is given on the Y-axis. Asterisks indicate the alleles that were Sanger sequenced (Genbank MN646783, MN684168, MN684204). The markers are displayed in the order of the color of the fluorescent label used (FAM, NED, PET), and size order, as is the case in Table 1.

*The motif is indicated by the sequence found in TRF track in the UCSC Genome Browser. The TRF track follows the nomenclature recommendations of the DNA Commission of the International Society of Forensic Genetics better than that of RepeatMasker (21, 22). Numbers for the motif sequences are from the horse reference genome, and bold underlined letters are not included in the repeat count (see Figure 1)*.

*The reference genome size (ref size) includes the additional non-templated adenine that is attached via the terminal transferase activity of the Taq polymerase (20)*.

*The concentration is the final value used in our implementation of the multiplex panel*.

*The stutter is given in the percent of the height of the main peak. The number for each marker is the average stutter across all measurements*.

*mut rate, This is the predicted mutation rate based upon the relationship of the SW score with the human mutation rate, assuming that the horse mutation rate will be similar (Venta, unpublished results). The mutation rate for Eca04a (ECA04.104.2) is likely to be too high because much of the repeat region is imperfect and it is very long (see Discussion)*.

a *SWS, Smith Waterman score, as obtained from the UCSC Genome Browser for equCab2*.

b *wt avg, weighted average in base pairs, as is the range*.

### Exclusion Analysis

In order to test the hypothesis that one of several candidate genes underlies the megaesophagus phenotype in Friesian horses, tetraSTRs were identified flanking these genes. The candidate genes selected were *AAAS, COL4A5, COL4A6, FH, GDNF, HMGA2, NOS1, NOS2, NOS3, KIT, MED12, PTPN22, RASSF1, RET, SPRY2, SPRY4*, and *VIPR1*. Tetra- or pentaSTRs were identified using the UCSC Genome Browser and SW scores above 450. Primers were designed to amplify these repeats using Primer3 (http://bioinfo.ut.ee/primer3-0.4.0/). Primer sets were then examined in DNA samples from affected and unaffected Friesian horses, as well as samples from eight horses of other breeds (Appaloosa, Arabian, Belgian, Quarter horse, Miniature horse, Percheron, Thoroughbred, and a mixed breed horse). Amplicons were labeled with a fluorescently labeled M13 tag sequence as an inexpensive way to implement high resolution genotyping (see below under Universal primers).

### The Primer Set for Sex Determination in the Amelogenin Gene

Sequences were aligned from Genbank records AB091793.1 (amelogenin sequence on the horse X chromosome) and AB091794.1 (amelogenin sequence on the horse Y chromosome) using the SeaView alignment software (21). Regions were selected surrounding a gap in the Y sequence compared to the X sequence and for which primers exactly matched both the X and Y chromosomes. Primer sequences are shown in Table 1.

### PCR Amplification

Primer sets were initially amplified as single amplicons using the following conditions: 10 mM Tris (pH 8.3), 50 mM KCl, 1.5 mM MgCl_2_, 0.1 mM dNTPs, 0.02 U Amplitaq (Qiagen, USA) per μl of final reaction volume and ~10 ng horse DNA (quantified by nanodrop and Qubit) in a 25 μl reaction. Cycling conditions were 1 min 94°C, 2 min 59°C, and 3 min 72°C for 50 cycles. Potential variability was assessed by running a 2% agarose gel and examining allelic and heteroduplex bands from a sample of Friesian and other horse breeds. For multiplex reactions, two sets of conditions were used. The first set was 10 mM Tris (pH 8.3), 50 mM KCl, 2.0 mM MgCl_2_, 0.2 mM dNTPs, 0.04 Amplitaq Gold (Qiagen, USA) per μl, a primer mix to produce the final concentrations given in Table 1, and 10 ng horse DNA in a 25 μl reaction. Multiplex cycling conditions were the same as for single amplicons, except that the annealing temperature was 57°C. The second set of conditions used the Multiplex Microsatellite Type-It kit (Qiagen, USA) (12.5 μl) with 4.8 μl of primer mix and 2 μl (10 ng/μl) of DNA. The cycling conditions were 5 min 95°C for the initial denaturation and hot-start Taq activation step, then 30 s 95°C, 1.5 min 60°C, and 1 min 72°C for 40 cycles, and final extension at 68°C for 90 min. Primer concentrations were adjusted to bring the peak heights for all markers to an acceptable balance (generally less than a three-fold difference among different markers as assessed by homozygous peak height ratios). Cross-species amplifications (the species are given above under “DNA samples”) were also examined with no horse DNA included and amplified under the same conditions for the final multiplex panel.

### Universal and Directly Labeled Primers

In order to reduce the expense of testing markers for variability, several universal primers were used. These included the M13 primer, and primers C and D of Blacket et al. (22). After we discovered that at least the M13 universal primer caused primer-dimer among some horse-specific primer sets, a switch was made to universal primers that had the dinucleotide AA at the 3' end (Supplementary Table S2) (26). Because primer-dimer was a significant problem in the multiplex panel with the original primers that were already directly labeled, many reverse primers were redesigned to have AA at the 3′ end.

### Multiplex Panel Genotyping

Two alleles for each marker were chosen for Sanger sequence analysis. This was accomplished either by using the amplification primers if they were far enough away from the STR to allow the complete repeat to be sequenced, or by designing new primers that were further away from the repeat (Supplementary Table S2). Sequences were aligned with the reference genome using the SeaView software package and the repeat numbers were counted (21).

### Nomenclature

STR loci were named according to their approximate chromosomal base locations based upon the Broad/equCab2 assembly, although for brevity in the text, they are generally just designated by the particular chromosome upon which they reside. Motifs were based on the upper strand (all markers show the same strand orientation in the reference genome builds EquCab 2 and 3). Repeat motifs were based upon the designation by Tandem Repeats Finder (TRF) as given in the UCSC Genome Browser (27). We have found these reported motif sequences to conform well to the recommendations published by the DNA Commission of the International Society for Forensic Genetics (28, 29). The two alleles for each marker were bidirectionally Sanger sequenced and the repeat numbers were counted. Alleles were then named by the number of repeats sequenced. Repeat numbers for the other alleles contained in this report were inferred from this data.

### Stutter Analysis

Stutter peaks were compared to allele peaks to determine stutter rates. Ten peaks were analyzed per marker. Alleles were only used if they were homozygous, or heterozygous and for which the two alleles were more than one repeat apart, and if the allele or stutter peak did not coincide with another peak that had a pull-up peak of the same color.

### Genetic Analysis Using Genalex and Cervus Programs

After manual correction of rounding errors from main peak sizes, alleles and genotypes were analyzed using the Genalex analysis package (30, 31). For example, output from the high-resolution genotyping system using the local Southern method might give 100.48 base and 100.52 base alleles for different horses, which are both considered to be the same allele (and they were manually corrected to be the same allele). Only 16 markers for this analysis were used because it was discovered after the analysis of all of the horses that one marker (Eca28) had apparent null alleles in more than 50% of the Quarter horses, Thoroughbreds, and Paints. After the main analysis, these primers were redesigned and the new designs worked in all horses (those primers are reported in Table 1). The Cervus program was also used to check the power to exclude for parentage (32).

## Results

### Development of a 17-Plex Plus an Amelogenin Marker

A 17-plex panel of horse tetra- and pentaSTRs was developed, plus an amelogenin (amel) marker that can be used to distinguish the sex of the horse (Supplementary Figure S1). The average number of alleles per marker was 8.3 ± 2.3. The average observed heterozygosity (Ho) and expected heterozygosity (He) for all markers for different breeds can be found in Table 2. Allele frequency distributions are shown in Figure 1. The probability of identity (PI; the probability that two genotypes from unrelated horses will match at random) within a given breed ranged from 1.1 x 10^−11^ to 1.5 X 10^−12^ and the PI among siblings (PIsib, the chance complete matching of genotypes of siblings) ranged from 1.4 x 10^−5^ to 7.2 x 10^−6^. These values were calculated for Paint, Quarter horse, Standard bred, Thoroughbred, and Arabian horses, where sample sizes of 10 or greater were available. Other breeds were genotyped, but the sample sizes were insufficient to produce meaningful calculations.

**Table 2:** Genetic information for markers in the 17-plex panel.

**Marker**	**Ho**	**He**	**He SW**	**PE1**	**PE2**	**PI**	**PIsib**	**Motif unit**
amel (XY)	0.56	0.68	NA	NA	NA	NA	NA	2
Eca28	0.80	0.79	0.71	0.579	0.401	ND	ND	4
Eca15	0.75	0.80	0.74	0.442	0.281	0.06	0.36	4
Eca07	0.64	0.75	0.71	0.639	0.461	0.12	0.42	4
Eca05	0.59	0.73	0.72	0.675	0.498	0.16	0.46	5
Eca12	0.56	0.69	0.75	0.726	0.561	0.18	0.47	4
Eca24	0.79	0.74	0.72	0.631	0.452	0.12	0.42	5
Eca22	0.60	0.74	0.73	0.591	0.411	0.14	0.45	4
Eca21	0.53	0.62	0.74	0.768	0.595	0.27	0.56	4
Eca25	0.55	0.62	0.74	0.791	0.650	0.31	0.56	4
Eca27	0.60	0.65	0.74	0.764	0.608	0.24	0.51	4
Eca14	0.63	0.63	0.61	0.771	0.607	0.23	0.51	4
Eca04a	0.68	0.73	0.75	0.668	0.488	0.13	0.44	4
Eca04b	0.69	0.74	0.66	0.664	0.488	0.14	0.44	5
Eca02	0.69	0.77	0.75	0.589	0.412	0.12	0.41	4
Eca03	0.66	0.75	0.73	0.657	0.481	0.13	0.42	4
Eca01	0.63	0.69	0.75	0.662	0.475	0.14	0.44	4
Eca20	0.89	0.86	0.75	0.426	0.270	0.03	0.32	4
Avg	0.66	0.72	0.72	0.650	0.479	0.158	0.449	
combined				9.11E-9	1.11E-5	2.0E-14	2.3E-06	

*The entire horse panel was used in this analysis (combined values are lower for individual breeds; see the text). Sort order is the same as in Table 1. Ho is the observed heterozygosity, He is the expected heterozygosity, He SW (Smith Waterman) is the expected heterozygosity based upon comparison to the human SW scores, assuming a maximum heterozygosity of 0.75 [Venta, unpublished results; the heterozygosity is based on the maximum diSTR heterozygosity for the majority of purebred horse breeds (11)], PE1 is the probability of parentage exclusion when both parents are available, and PE2 is the probability of exclusion when one parent is missing (Cervus output), PI is the probability of identity (probability that two genotypes from unrelated horses will match at random) and PIsib is the probability of distinguishing individual siblings among themselves (Genalex output). Amel data are based upon the allele frequencies that are present in the X chromosome amplicon*.

Although there was some weak ability to identify horse breeds based upon the output of Genalex analysis (mean correct assignment 65%), some horses clustered with the wrong breed. One marker, Eca12, had some tendency to produce split peaks, despite the presence of a pigtail on the reverse primer so, in addition to a prolonged final extension, some care was necessary to infer the genotype. The multiplex panel produced good results with both blood and hair-root derived DNAs (data not shown).

Genalex identified six markers in the horse 17-plex panel that could potentially contain null alleles [based on Hardy-Weinberg equilibrium (HWE) calculations]. Those markers that were not consistent with HWE across horse breeds, were three markers (Eca05, Eca21, and Eca22) not conforming to HWE in two breeds and the other three markers (Eca01, Eca02, and Eca03) in one breed each (data not shown).

### Micro-Variants

Relatively rare micro-variants (<2% of all alleles for a marker) were found in four of the markers: Eca02, Eca15, Eca20, and Eca25. These micro-variants were visually confirmed using the “overlay all” function of the Peak Scanner software, but were not sequenced in this study because of their rarity. A single micro-variant in the Eca12 marker was at a much higher frequency, 38% of all alleles. This variant was sequenced and found to be due to a single base indel (G/-) outside of the repeat, near the edge of a poly-T run of 10 bp (the location can be seen by comparing Genbank records MN684185 and MN684186). It may be a good practice to bin the 17.3 microvariant for Eca12 with the nearest whole repeat (18) because the single base deletion is outside of the main repeat. This was suggested for certain microvariant alleles in the Mini-Dogfiler panel for the same reason (33). The frequency of these micro-variants, as well as the on-ladder alleles for all of the markers, are shown in Figure 1.

### Low Variability With Some of the Original Markers Near Candidate Genes in Friesian Horses

Some of the original horse primer sets used to examine candidate genes had low or no variability within the unaffected Friesian horse population, despite having SW scores that are correlated with heterozygosities higher than 65% in purebred dogs and 86% in human outbred populations (Venta unpublished results; Supplementary Table S1). This demonstrates that, at least within some mammalian genomes, in order to have reasonable variation it is first necessary to have an uninterrupted perfect repeat (also known as LUS) of at least 8, no matter how high the SW score for the particular microsatellite (25). One exception was a marker called MK21 (Supplementary Table S1). Although the longest uninterrupted tetranucleotide repeat was three for the MK21 primer set, this marker had a longer perfect repeat that was 16 bases long and was repeated five times in the reference genome. Thus, on occasion, a tetraSTR (as identified in RepeatMasker) may have variation despite not having an LUS of eight or more for the tetraSTR, and its variability may depend upon higher order repetitions within a given marker. We have observed this for a few other markers in other species as well (unpublished results).

### Occurrence of Strong Primer-Dimer Formation When Using an M13 Universal Primer and Employment of the “AA” Strategy

An examination of several markers for amplification showed that the M13 primer that we used to determine the variability of markers could cause strong primer-dimer formation (Supplementary Figure S2). For this reason, a new set of universal primers was developed that reduced the probability of primer-dimer formation. This set of universal primers was used in previous studies for other species (28, 34). The basis for reducing primer-dimer formation was the design of most primers with AA at the 3′ end (35).

### Observations for Specific Primer Sets

MK21, in addition to showing reasonable heterozygosity despite having an LUS of only 3 for the tetraSTR (as described above), also migrated aberrantly compared to its sequenced size. It was therefore not included in the final multiplex panel. It is not clear what causes the fragments to run dozens of bases too quickly, but it seemed prudent to exclude it from the multiplex panel. Another marker called 7981, which is located on chromosome 6, was excluded from the multiplex panel because different alleles either did or did not include a deletion of 12 bp outside the primary repeat. Marker 8019 on chromosome 11 was excluded because the repeat structure is complicated and would have caused difficulties in nomenclature. Marker Eca28 in the final 17-plex panel required a redesign because the original set failed to amplify in many horses, mostly within three breeds (Arabian, Thoroughbred, and Paint). The redesigned set, included in the multiplex panel, no longer had the null phenotype.

### Stutter

Stutter for the markers ranged from 1.2 to 11.3% (Table 1). The highest stutter was found for the marker Eca20, ~11% stutter averaged across all alleles, which had the largest LUS in the reference genome (18), and also had the largest number of observed alleles (17) among all markers in the 17-plex panel.

### Cross-Species Amplification

DNA samples from several other species were also subjected to PCR with the 17-plex panel. Zero to four small peaks (<200 bases, except for the goat) were observed among the different species examined (Supplementary Figure S3). Based upon the published results for other species, this result does not seem to be unusual, but it should be taken into account if these markers are used for certain forensic analyses (33).

### Friesian Candidate Gene Exclusion Analysis

Markers within 2 Mb of candidate genes were examined in a group of 13 Friesian horses that had been diagnosed with megaesophagus. If a marker showed heterozygosity for two alleles that differed by more than one repeat unit, the corresponding gene was considered to be excluded as underlying a recessive mode of inheritance for megaesophagus; for discussions of criteria and assumptions underlying exclusion analysis (6, 36). If homozygous markers were not shared among all affected horses (i.e., if they were homozygous for different alleles in different affected horses), they were excluded under a dominant or X-linked mode of inheritance. Results for excluded genes are shown in Supplementary Table S3.

## Discussion

We have developed a 17-plex panel of 14 tetra- and 3 pentaSTRs for the horse, plus a sex-discriminating amelogenin marker. All but 2 of these markers are on separate chromosomes. The two markers on chromosome 4 are more than 68 Mb apart in the reference genome (inferred to correspond to >50 cM), and are therefore assumed to assort independently. This situation is similar to the human CODIS markers D5S818 and CSF1PO, which are both on chromosome 5, separated by 26.4 Mb and which appear to independently assort (https://strbase.nist.gov/fbicore.htm). Across all markers, the average Ho was 0.66 and the average He was 0.72. To produce a cost-efficient tool, we used HEX, FAM, and NED fluorescent labels. If a five-color system is desired (e.g., with FAM, NED, PET, and VIC, with LIZ for the size standard), it would be relatively simple to make adjustments, with the caveat that the various dyes may cause slight differences in migration (37). Five of the markers had relatively rare microvariants, which is similar to the frequency of microvariants in the human CODIS panel (38, 39). Genalex identified six markers in the horse 17-plex panel that could potentially contain null alleles (based on Hardy-Weinberg equilibrium [HWE] calculations). Sample sizes larger than examined here (the 10-18 horses in the five individual breeds) are needed to determine if noteworthy null alleles may exist for given breeds for any of the markers.

This horse 17-plex panel should be useful for both profiling and paternity testing purposes. The determined Ho, He, PI, and PIsib were similar to the ranges found for diSTR multiplex panels currently in use for genetic profiling of the horse (Table 2) (11, 40). It would also be possible to develop a marker ladder for horse tetraSTR multiplex panel, which would make automated genotyping and cross-laboratory comparisons for horses more reliable. The disadvantages of diSTRs, particularly with respect to forensic applications, have been discussed before and because the new horse 17-plex nomenclature is based upon the number of repeats rather than letters, these disadvantages are largely eliminated (41). The sex-discriminating amelogenin marker includes a diSTR (on the X chromosome only) that could also be used to marginally increase profiling significance, if desired.

Our current data set is too small to identify mutation rates for this set of markers, but we have provided an estimate based upon the relationship between SW scores and mutation frequencies in humans (Table 1; Venta unpublished results). These predicted mutation rates (average of 0.4 mutations per 100 meioses per marker) are higher than those seen among the human CODIS markers (the CODIS mutation range is 0.012-0.189 per 100 meioses; http://www.cstl.nist.gov/biotech/strbase/ mutation.htm). Regardless, we believe that they are within an acceptable range for most laboratories that will use the panel (Table 1). To our knowledge, the mutation rates or estimates of the mutation rates for the equine diSTRs currently in use have never been reported. We speculate that the predicted mutation rate for one marker (Eca04a) in the 17-plex panel in this report is too high. The speculation was made under the assumption that the type of mutational mechanism that occurs among tetraSTRs is similar to the model put forth by DN Cooper and colleagues, in that sequences within ~25 bp of a mutation site seem to be the ones with the greatest influence on the production of a new mutation, which occurs most often in the LUS of STRs (42, 43). However, future experience with these markers will determine how close the predicted mutation rates will be compared to the future observed mutation rates.

SNPs are also being examined for use in horse profiling (16, 17). However, several issues need to be addressed before they are put into common use. These include the choice of SNPs to be used and how to handle linkage disequilibrium. Many more markers are needed to have the same power as STRs. Marker spacing may therefore be such that they may be close enough to each other to cause concern for potential confounding due to co-segregation of marker alleles from neighboring SNPs. Lastly, there is the cost of implementing the new technology in individual labs. The panel reported here avoids these issues and can be implemented relatively quickly by those laboratories already using diSTRs, and with a similar power to the currently used, and similar-sized, multiplex diSTR panels.

The use of SW sequence alignment scores is quite helpful for identifying tetraSTRs markers that have good genetic variability and predicted reasonably low mutation rates [(28, 34), current report]. TetraSTRs with only perfect long uninterrupted repeats can be used to identify variable markers, but they have either low genetic variability (if the LUS is <8 repeats) or high stutter (more than 16 LUS repeats) compared to markers that have been found to be most useful [e.g., the CODIS markers, (25)]. However, as shown by the markers used for exclusion analysis of the candidate genes for Friesian megaesophagus, it is necessary to have an LUS of at least eight to have acceptable variability. Markers with SW scores even hundreds of points above 500 will have little variability unless they also have a reasonably long LUS (Supplementary Table S1). Markers with a moderately long LUS and with some flanking region imperfect repeats will have good variability, reasonably low stutter, and relatively low mutation rates if the SW scores are kept within a narrow window (e.g., 450-700 using the original RepeatMasker scoring system available in the UCSC Genome Browser before around 2012).

The use of universal markers helps to reduce the cost and development time of markers. Although some universal markers are already present in the literature (e.g., universal primers based upon M13 bacteriophage sequences), we found that some primer sets will produce only strong primer-dimers when attached to about 20% of the species-specific primers, and so we produced a new type of universal marker for preliminary examination of markers (28, 34). These markers are based upon the use of AA at the 3′ end of the primers. It is, therefore, necessary to find primer sequences that end in AA in the target genome, which is a limitation of the method. However, the method makes it possible to produce multiplex panels as large as 18 with a minimum of primer re-design (35, 44). Although it is not necessary to have all primers end in AA, it has been our experience that the larger the percentage of such primers are in a multiplex will lower the probability of primer-dimer formation. Although the whole system requires more work early in the choice of STRs and primer design stages, we believe it saves considerable time and effort in the work required later in the process of developing multiplex panels. This can also be quite important in species such as the horse where tetraSTR choices are limited.

Sequence analysis of two alleles from each marker was consistent with expected allele numbers for the markers in the 17-plex panel. Three markers with reasonably variability that we had originally planned to be included in the panel were discarded because of (1) aberrant migration (Eca08, referred to in this report by the primer set name MK21), (2) a polymorphic 12-bp indel outside of the repeat interval that would cause difficulty with eventual nomenclature (Eca06, primer set 8019), or (3) very complicated repeat structures that could have led to nomenclature complications (i.e., Eca11), demonstrating that it is always worth developing a few more markers than desired for a final set (Supplementary Table S2).

This 17-plex panel will probably find its main uses in profiling and paternity testing in horses. Nonetheless, it is worth mentioning some other potential uses of it. Some experimental work is currently being done in horse cell lines which may be used in investigations of pathology (45). In human cell line work, a significant number of cell lines have been misidentified, resulting in irreproducibility in the scientific literature (46). The markers reported here can be used to verify that the correct horse cell lines have been used, possibly using the new universal primers for labeling, mentioned in the methods section, and a subset of the full 17-plex panel in order to speed the process and limit the cost. In addition, the primers may serve as a foundation for the development of genetic identification systems of related equids, such as the donkey and zebra. We also note for those who wish to use some of the methods reported here for other species, that each species seems to have its own unique challenges for tetraSTR development [e.g., horse has a limited number of good tetraSTRs, zebrafish appears to have many null alleles, and the honeybee may require a longer LUS for good variability (46), this work, and Venta, unpublished results].

Finally, we return to the study that instigated the development of this 17-plex panel—that is, Friesian horse megaesophagus. In addition to the difficult choice of candidate genes for this poorly understood condition, and the initial difficulty of identifying tetraSTRs that have good variability near identified candidate genes, there is also the problem that the Friesian horse had an extremely tight bottle neck during and after World War I, which caused the breed to lose genetic diversity (47). The Friesian breed shows the lowest diSTR heterozygosity among 45 breeds studied worldwide (11). Five genes were excluded as being potentially causative in our analysis (*COL4A4, GFNF, PTPN22, RASSF1*, and *KIT* genes, under an X-linked or recessive model) but, unfortunately, we did not find a gene that was likely to be causative using exclusion analysis (Supplementary Table S3). It will probably be necessary to use a method that is more costly in terms of techniques and researcher's time, such as GWAS and/or whole genome sequence analysis. Nevertheless, we hope ultimately that the underlying genetic basis can be found so that it will be possible to remove this undesirable condition from this magnificent breed.

## Data Availability Statement

The datasets presented in this study can be found in online repositories. The names of the repository/repositories and accession number(s) can be found in the article/Supplementary Material.

## Ethics Statement

The animal study was reviewed and approved by Michigan State University IACUC. Written informed consent for participation had been previously obtained for the Friesian horse samples for testing candidate genes for megaeosophagus. Other horse breed samples were leftovers from a previous study on another heritable condition. Permission to release genotypes that could identify the individual horses was not obtained, but summary statistics are provided. Sequences are in Genbank.

## Author Contributions

MKo, IL, MKi, and PV: conceptualization. SE and TT: resources. AL, MKo, TC, TT, and PV: methodology. PV: writing—original draft. AL, MKo, TT, TC, SE, MKi, IL, and PV: writing—review and editing. All authors contributed to the article and approved the submitted version.

## Funding

This work was funded by the MSU College of Veterinary Medicine Endowed Research Funds, Freeman Fund for Equine Health, and the MSU Veterinary Diagnostic Laboratory.

## Conflict of Interest

The authors declare that the research was conducted in the absence of any commercial or financial relationships that could be construed as a potential conflict of interest.

## Publisher's Note

All claims expressed in this article are solely those of the authors and do not necessarily represent those of their affiliated organizations, or those of the publisher, the editors and the reviewers. Any product that may be evaluated in this article, or claim that may be made by its manufacturer, is not guaranteed or endorsed by the publisher.
