# Supplementary material for: Development of a 17-Plex of Penta- and Tetra-Nucleotide Microsatellites for DNA Profiling and Paternity Testing in Horses
Source: Front Vet Sci. 2022 Apr 7;9:861623. doi: 10.3389/fvets.2022.861623 (PMC9021955; doi:10.3389/fvets.2022.861623)
Supplement: Supplementary file 1 [file Data_Sheet_1.PDF]

Luttman et al., supplementary Tables, Figures, and information.

Table S1. Marker observations for LUS and Smith-Waterman scores vs. heterozygosity using Friesian horse candidate gene markers.

marker no.	LUS	SWS	Ho	He	SWS het	motif	c'some	position	F primer	R primer	notes	gene
MK29/30	2	503	0.00	0.00	0.683	(CATA)n	chr4	102599688	AAACGCTCCCTTGATAGCAG	TTACCACACTTTGTTTAGGATG	gel	*NOS3*
MK31/32	3	479	0.00	0.00	0.663	(CATA)n	chr4	102599688	CCTCAGAATGCTTTCATTCATC	ATTCTGGTCCCTTCCTCTCC	gel	*NOS3*
MK49/50	3	621	0.00	0.00	0.733	(TCTA)n	chr17	52587882	CCAAATTGAATAAAAGACTGCCTA	TGCTTGAGCTATGCATTCCA	gel	*SPRY2*
MK21/22	3	719	0.35	0.40	0.745	(TGGA)n	chr8	16092028	TGCATGAATTGAAATGGACAC	TTGATTACTCCTTGGCACACC	123 horses	*NOS1*
MK27/28	4	787	0.00	0.00	0.748	(TCTA)n	chr11	41905994	ACTTGGGCCATCTTGAATTG	CAAAGACACATACACACCCACAC	gel	*NOS2*
MK45/46	4	365	0.00	0.00	0.492	(TTTC)n	chr1	71057601	TGGTTTTGAAAGTTTTCCTCTGA	GCATGAACAGACAAGCAATGA	gel	*RET*
MK63/64	4	447	0.00	0.00	0.629	(TCTA)n	chr30	3160458	ATTGGGCTAATGCAAAGGAA	AAGAGGAAAGATGAAGAGAATCATAAA	gel	*FH*
MK09/10	5	449	0.00	0.00	0.631	(TCTA)n	chr6	70280598	AGTGTGACAACCGACAGGTG	CTGGCCCATGCTAAGTACAC	gel	*AAAS*
MK15/16	7	441	0.50	0.66	0.621	(TCTA)n	chr5	54868236	GAAGGACTCTCCTGGATCTGG	GAAATATGTTAAAATCCCATGTTTGA		*PTPN22*
MK35/36	7	396	0.25	0.23	0.552	(TCTA)n	chr3	77727879	CGTTACATCATATTGTCCTGCATC	TTAAAGCCCTGTCACCCAGT		*KIT*
MK19/20	8	419	0.25	0.54	0.590	(TTTTA)n	chr16	36932857	CCTCTGTACTGAAATGGAAATGAC	AGTGGTGTACCCACAACATGAC	pentaSTR	*RASSF1*
MK41/42	9	486	0.75	0.72	0.670	(TCTA)n	chr21	27741678	CCTCAGGTTAAGGCATCCAC	CAACTGCCTTGTTGACATATCC		*GDNF*
MK11/12	10	833	0.67	0.60	0.749	(TCTA)n	chr6	70280598	TGTTAGTAGGGAGTAGGGAGTGG	GGGAGTTGGCGTTATTTAGG		*AAAS*
MK33/34	11	396	0.86	0.58	0.552	(TTTA)n	chr3	78537162	CCTCCTTTCCCAGAGACTCC	TGTAATAATGTATGCCCGAGATTTAC		*KIT*
MK13/14	14	540	0.25	0.53	0.706	(TTTC)n	chr5	54868236	TTCATGTAGCAGGCTTGTGG	CATTAAACAATGCATGACTGTAATAGG	strong PD	*PTPN22*

LUS = longest uninterrupted sequence (Brookes et al., 2012).

Except as noted, all Ho (observed heterozygosity) and He (expected heterozygosity) are based on genotypes for 8 horses, one each of the following breeds:

Appaloosa, Arabian, Belgian, Quarter horse, Miniature, Percheron, Thoroughbred, and mixed breed.

SWS score heterozygosities (SWS het) were based upon the logistic curve (Venta et al. unpublished result) with a maximum asymptote (heterozygosity) of 0.75.

Observed heterozygosity for half of the markers are based upon agarose gels that show no variability (indicated under the notes column; heterozgotes can be detected by heteroduplex bands even for single step allele differences); all other samples are high resolution genotypes.

All of the F primers included an M13 sequence tag for labeling of the amplicon (the sequence is in Table S2; Schuelke, 2000).

Strong PD = extensive primer-dimer formation.

Markers used for exclusion analysis that are diSTRs, located on the X chromosome, or that did not amplify (possibly because the M13 primer induced significant primer-dimer formation) are not shown.

MK21 has an LUS for the tetraSTR of 3, but a longer 16 bp repeat that has an LUS of 4 in the reference genome. Interestingly, alleles for MK21 differed in size by 4 bp.

Note that, except for MK21, markers only show variability at or above an LUS of 7, regardless of the SWS.

PD = primer-dimer

Table S2. Sequencing and other primers used in this study that are not shown in Tables 1 or S1.

target	primer name	sequence	ref size	purpose	reference
	M13	AGGGTTTTCCCAGTCACGAC	NA	universal primer	Innis et al., 1988
	Blacket C	CAGGACCAGGCTACCGTG	NA	universal primer	Blacket et al., 2012
	Blacket D	CGGAGAGCCGAGAGGTG	NA	universal primer	Blacket et al., 2012
	uni-1	CTCCAACTCACCTCCAACAAA	NA	universal primer	Corner et al., 2018
	uni-2	AAACCTCTCTCCACACCCAAA	NA	universal primer	Corner et al., 2018
	uni-3	CTCACCTCCCACTCCACAAA	NA	universal primer	Corner et al., 2018
	uni-4	AACTCCACCACTCCCACAAA	NA	universal primer	Corner et al., 2018
Eca06	7981 (F)	AGGCCTTATATGCATTGCAGAGTG	212	abandoned tetraSTR	this report
Eca06	7982 (R)	gTGGACTCTTTTCACTGTCTGACTAAA		abandoned tetraSTR	this report
Eca11	8019 (F)	ACTGGTCCTGCCTGTCTCTG	383	abandoned tetraSTR	this report
Eca11	8020 (R)	TTGAACTCTAGTGGTGCTAAGATTG		abandoned tetraSTR	this report
Eca22	SQ-8124	GATTTTGGCATTGTGCAAAC	321	allele sequencing	this report
Eca24	SQ-8168	TGAAAAGAGTGCACTTGAGGAA	349	allele sequencing	this report
Eca04b	SQ-8294	GAGTCTGTTTCCTTGGTTTTCCTA	272	allele sequencing	this report
Eca02	SQ-8224	GAGAACGGAATGAGTTGTTGAA	277	allele sequencing	this report
Eca28	SQ-8325	AAGAATGCCAGCTAACAGAAAA	250	allele sequencing	this report
Eca06	SQ-7981	TTTCCACTTTCACTATCTTTGGA	379	allele sequencing	this report
Eca06	SQ-7982	CGATGGTAGCAAGTTGTTGAAA	379	allele sequencing	this report

Eca15	SQ-8073	TGCTTGGTGTACAGGCCTCAG	243	allele sequencing	this report
Eca07	SQ-8283	GAACAATGAGCAGGGAGTACAA	289	allele sequencing	this report
Eca05	SQ-8159	GGACTTTCAAAACTCACCCAAA	346	allele sequencing	this report
Eca11	SQ-8019	CTACTGGTCCTGCCTGTCTCT	389	allele sequencing	this report
Eca12	SQ-8317	AAGGAGCAAGTTCAGGCAAA	432	allele sequencing	this report
Eca25	SQ-8321	TCTGAGAGGTGATGGCAAAA	246	allele sequencing	this report
Eca14	SQ-8319	AATTTCTAAAAGGGCACAGGAA	400	allele sequencing	this report
Eca04a	SQ-7997	CCCTATTCATCATTGAGGGTTT	529	allele sequencing	this report
Eca03	SQ-8225	CAGGGTTTTCTACGAGTTGGA	375	allele sequencing	this report
Eca01	SQ-8047	TTTGGAGATGTTGGAAGTTAAGG	294	allele sequencing	this report
Eca02	SQ-8223	CCCAAGGAGTATTGTTGAGACA	308	allele sequencing	this report
Eca08	SQ-MK21	AGAAAAATGCCGAAAAACTCC	342	allele sequencing	this report
8123	SQ-8123	TTTTGTGTGGCACTGCTGATAA	362	allele sequencing	this report

ref size = Amplicon size in the horse reference genome, Equcab2.

For primers with 4-digit numbers, odd number primers are forward (F) primers and even numbered primers are reverse (R) primers.

For primers used for sequence analysis (SQ) they should be paired with the appropriate F or R primer from Table 1, unless there is a partner in this table.

NA = not applicable

Table S3. Friesian horse data for exclusion of five candidate genes.

gene	COL4A6		GDNF		KIT		PTPN22		RASSF1	
primers	MK7/MK8		MK41/MK42		MK33/MK34		MK15/MK16		MK19/MK20	
chromosome	X		21		3		5		16	
SWS	399		486		396		441		419	
LUS	9		9		11		7		8	
ref size	184		217		290		249		237	
motif	(TCTA)n		(TCTA)n		(TTTA)n		(TCTA)n		(TTTTA)n	
allele	1	2	1	2	1	2	1	2	1	2
affected horse										
1	165	173	213	213	287	287	226	226	209	220
2	165		213	213	294	298	218	230	214	220
3	165	173	209	213	294	294	226	230	204	220
4	165	161	213	213	287	295	226	230	220	220
5	173		213	213	294	294	218	226	220	220
6	173		213	213	294	298	226	230	220	220
7	173		208	208	294	294	218	226	203	220
8	164		214	218	294	294	226	230	220	220
9	164		213	213	294	298	221	229	219	219
10	164		208	217	294	294	217	229	203	214
11	172		214	214	294	294	225	229	214	220
12	164	172	213	213	294	294	218	226	209	219
13	164		208	213	287	294	ND	ND	219	219

Footnotes: Ref sizes include the FAM-labelled M13 tail (AGGGTTTTCCCAGTCACGAC; no pigtail was applied to the R primers). Boxed cells indicate the alleles that exclude the gene as causative under an X-linked or dominant model. Recessive exclusions are inferred from alleles that show heterozygosity for a given marker among the affected animals. Note that only affected animals are needed for exclusion analysis, under the assumption that a marker is variable in the population (Winkler et al., 2013; Te Meerman et al., 1995).

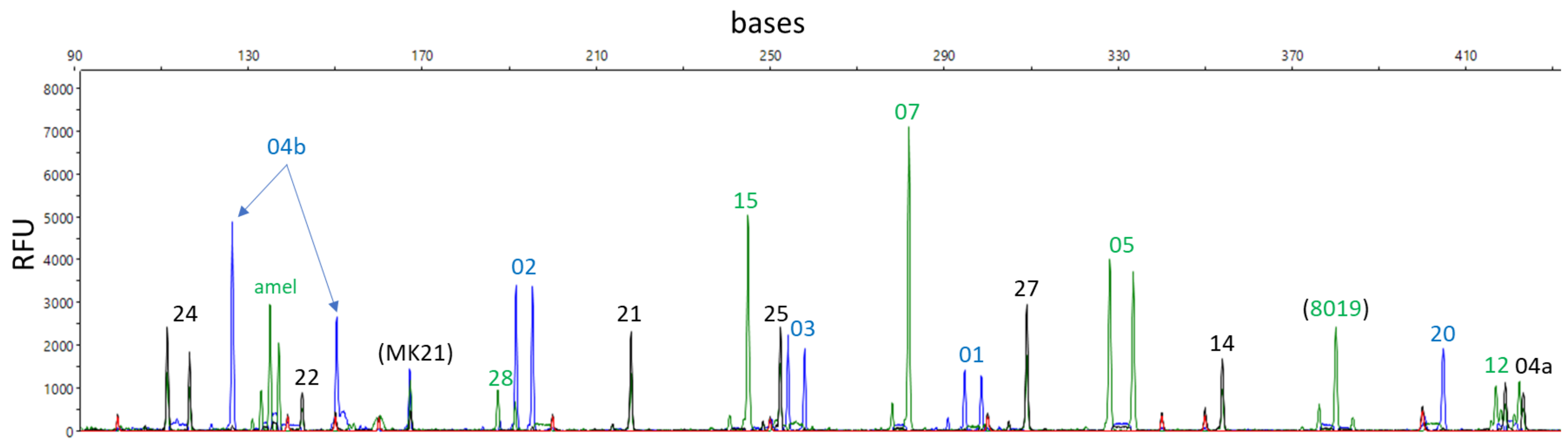

Figure S1. Representative 17-plex electropherogram. RFUs are the relative fluorescence units provided by the ABI 3730xl instrument. Peaks are labeled with the chromosome number upon which the marker occurs (Arabic numbers are colored to correspond to the correct peak), except for two markers (MK21 and 8019) which are abandoned markers no longer used in the multiplex (see the main text for a description of these markers). Some cross-channel fluorescent "pull-up" is seen among some of the markers, which may be reduced by optimization of the ABI 3730XL spectral calibration for these dyes. For this figure, the DNA was from pulled hair roots. FAM peaks are blue, HEX peaks are green, NED peaks are black, and the small red peaks represent the ROX-labelled size standards.

Supplemental material on the identification of the M13 primer as a likely cause for the lack of amplification of some tetraSTR markers.

During the develop of the tetraSTRs reported in this document we used the M13 primer along with the forward (F) primer (with an M13 tag) and the reverse primer (R) for our initial screening efforts. We noticed that about 20 to 25% of our primer sets did not amplify well (often accompanied with a significant primer-dimer band) and these primers were set aside. However, at one point we began to use only the F and R primers for the initial screen for amplification (0.2 μM final concentration for both primers, with all other conditions as reported in the primary report), without the inclusion of the universal M13 primer. Five of these markers all produced strong amplification products (data not shown). However, when we then went to amplify these markers with the inclusion of the M13 (FAM-labelled) primer, for one of the tetraSTRs, we were surprised to find no expected amplicon was produced, and only a primer-dimer band was observed (data not shown).

We then designed an experiment to test the hypothesis that the primer-dimer was caused by an interaction of the M13 primer with the F and R primers. A second M13 primer was designed that exactly matched the original primer except that AAA was added to the 3' end (Figure S3). A new F primer was designed so that both the old and the new M13 primer would anneal to its complement. Amplifications were then conducted with the original M13 primer, the modified M13 primer, and with regular Invitrogen Taq or Amplitaq Gold (a hot start Taq). Five horse DNAs were amplified along with a water control. With the regular Taq and the original M13 primer, a strong primer-dimer band was observed and no tetraSTR bands. With Amplitaq Gold, and the original M13 primer, a strong primer-dimer band was still observed, but some tetraSTR product was observed for some of the horse samples. The same result was also observed the new M13 primer and standard Taq, except that the primer-dimer was not uniform in size across all of the horse samples. When the modified M13 primer was used with Amplitaq Gold, tetraSTR bands were observed for all horse samples with little to no primer-dimer.

We interpret this result to mean that the original M13 primer in three primer systems (i.e., the universal primer system) leads to primer-dimer formation, at least for some primer sets. Although we have no direct evidence that the M13 primer causes primer-dimer in all of the tetraSTR sets that have been set aside, a survey of the literature suggests that this may indeed be a common problem, because many papers have used the M13 primer for development of

microsatellites and it is typical to see about a 20% or more “failure to amplify” statement (e.g., Paz-García DA et al., 2017; Vartia et al., 2014). We have also found it easier to produce multiplexes of 10 or more markers using this method (i.e., primers all ending in AA), and with far fewer single target amplification failures (Corner et al., 2019; Keven et al., 2019; and the present work on the horse multiplex). Although we claim to have conceived of the use of AA at the 3’ end of primers independently of Randall Saiki (see Innis and Gelfand, 1999), we were preceded by more than 20 years when the original idea was first developed (we discovered his work shortly after we began using the AA method routinely). A few other individuals have noted the method in their publications, but we have not seen this concept routinely used (e.g., Graham and Holland, 2005; SantaLucia, 2007). It is tempting to speculate that three primer systems are most prone to rare templating events between poorly matched 3’ ends of the F primer and the M13. Amplification of the rare event product would become a common event because the M13 tag would then, in this case, perfectly match the chimeric F primer template. Using primers that end in AA is, therefore, an attempt to prevent the initial rare templating event from happening. We note that, even when using the universal primers, primer-dimers can still occur, particularly if the universal primer concentration is too great (unpublished data). It may be necessary to titer the universal primer concentration if primer-dimer is observed.

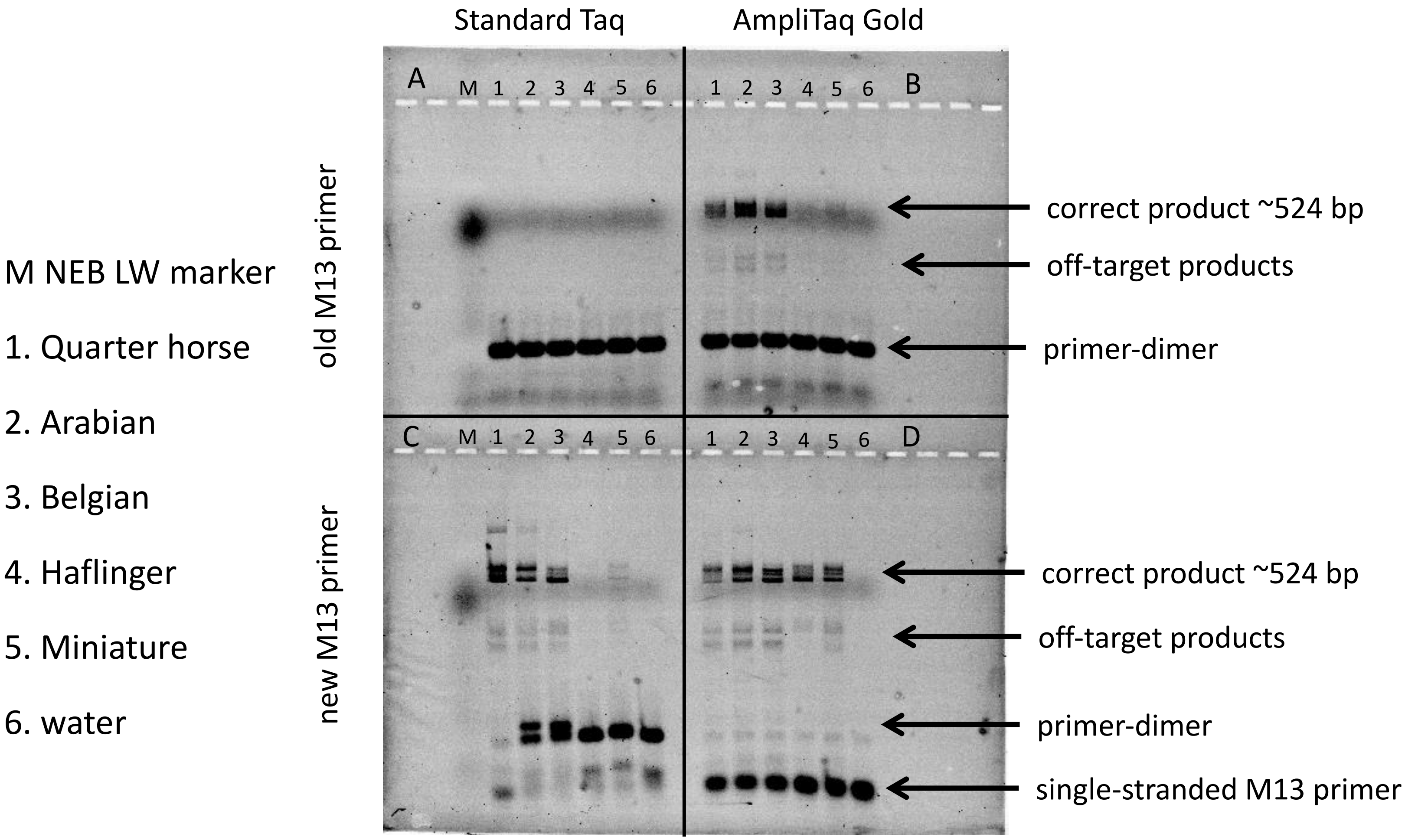

new M13: FAM-TGGTTTTCCCAGTCACGAC**AAA**

old M13: FAM-AGGGTTTTCCCAGTCACGAC

Chr18-F-aaa: AGGGTTTTCCCAGTCACGAC**AAA**CATATTCGCCTACCACCACTAAA Chr18-R: GAAATAAGGCAGTGAAAATGACAAA

Figure S2. Data suggesting that the M13 primer causes primer-dimer formation with some amplicons in the three-primer universal labeling system.

Both the original and a new M13 primers were tested using the first set of amplification conditions described in the article, except for the variables being examined. The new M13 primer sequence ends with AAA (underlined green letters). To keep the melting temperature the same between the new and original (old) M13 primer, AG was removed from the 5' end of the new primer and a T (blue) was added to prevent quenching of the FAM by what would have otherwise been the 5' G (Nazarenko et al., 2002). The gel was imaged with a Typhoon FLA 9500 instrument (GE Healthcare). A. Standard Invitrogen Taq polymerase with the old M13-FAM primer produced almost none of the desired amplicon. Virtually all of the fluorescent M13 primer went into primer-dimer. B. Amplitaq Gold polymerase with the old M13 primer allowed at least some label to be incorporated into the desired amplicon, but the amplicon for some of the horses is weaker than desired and most of the fluorescence is still in the primer-dimer band. C. The new M13 primer (ending in AAA) with standard Taq allows amplification of the correct product, but there are still primer-dimer bands (of apparently different sizes and presumably different composition for the different horses). D. When the new M13 primer and Amplitaq Gold are used in combination, all horse DNAs showed useful amplification of the desired product and primer-dimers are nearly completely absent. Presumably the primer-dimer bands in quadrant C are caused by interactions that occur only at low temperature (unlike those in quadrants A and B) because they are missing in quadrant D in which the hot start Taq was used. The marker examined in this experiment (on horse chromosome 18) was not used in the final horse multiplex because the amplicon size (>500 bp) was larger than desired. The size standards are not shown on this image, but were seen after ethidium bromide staining (data not shown).

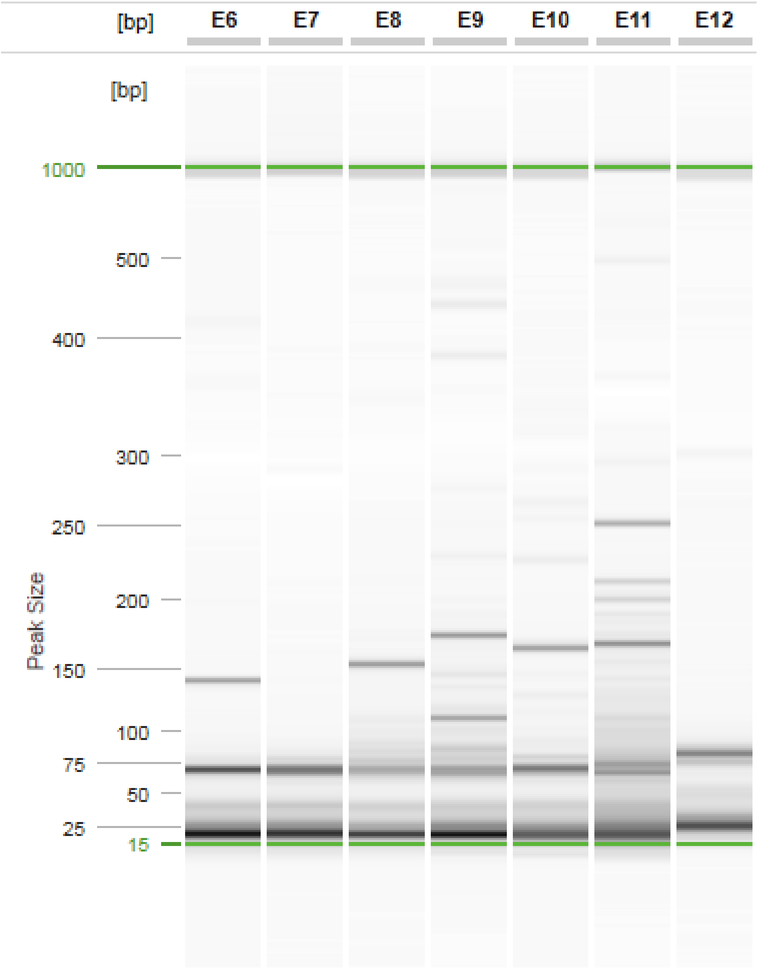

Figure S3. Small bands on cross-species amplification with other species with the 17-plex primer set. The species are E6, human; E7, mouse; E8, cat; E9, dog; E10, cow; and E11, goat. E12 is a water control. One or two bands of moderate intensity that are smaller than 200 bp appear for most species.

References for Supplementary Material

1. Bär W, Brinkmann B, Budowle B, Carracedo A, Gill P, Lincoln P, Mayr W, Olaisen B. DNA recommendations. Further report of the DNA Commission of the ISFH regarding the use of short tandem repeat systems. International Society for Forensic Haemogenetics. Int J Legal Med. 1997;110(4):175-6.

2. Blacket MJ, Robin C, Good RT, Lee SF, Miller AD. Universal primers for fluorescent labelling of PCR fragments--an efficient and cost-effective approach to genotyping by fluorescence. Mol Ecol Resour. 2012 May;12(3):456-63. doi:10.1111/j.1755-0998.2011.03104.x.

3. Brookes C, Bright JA, Harbison S, Buckleton J. Characterising stutter in forensic STR multiplexes. Forensic Sci Int Genet. 2012 Jan;6(1):58-63. doi:10.1016/j.fsigen.2011.02.001.

4. Brownstein MJ, Carpten JD, Smith JR. Modulation of non-templated nucleotide addition by Taq DNA polymerase: primer modifications that facilitate genotyping. Biotechniques. 1996 Jun;20(6):1004-6, 1008-10.

5. Corner S, Yuzbasiyan-Gurkan V, Agnew D, Venta PJ. Development of a 12-plex of new microsatellite markers using a novel universal primer method to evaluate the genetic diversity of jaguars (Panthera onca) from North American zoological institutions. Conserv Genet Resourc. 2019 Dec; 11(04): 487-497.

6. Graham KJ, Holland MJ. PrimerSelect: a transcriptome-wide oligonucleotide primer pair design program for kinetic RT-PCR-based transcript profiling. Methods Enzymol. 2005;395:544-53.

7. Innis MA, Myambo KB, Gelfand DH, Brow MA. DNA sequencing with Thermus aquaticus DNA polymerase and direct sequencing of polymerase chain reaction-amplified DNA. Proc Natl Acad Sci U S A. 1988 Dec;85(24):9436-40.

8. Keven JB, Walker ED, Venta PJ. A Microsatellite multiplex assay for profiling pig DNA in mosquito bloodmeals. J Med Entomol. 2019 Jun 27;56(4):907-914. doi:10.1093/jme/tjz013.

9. Te Meerman GJ, Van der Meulen MA, Sandkuijl LA. Perspectives of identity by descent (IBD) mapping in founder populations. Clin Exp Allergy. 1995 Nov;25 Suppl 2:97-102.

10. Nazarenko I, Pires R, Lowe B, Obaidy M, Rashtchian A. Effect of primary and secondary structure of oligodeoxyribonucleotides on the fluorescent properties of conjugated dyes. Nucleic Acids Res. 2002 May 1;30(9):2089-195.

11. Parson W, Ballard D, Budowle B, Butler JM, Gettings KB, Gill P, et al. Massively parallel sequencing of forensic STRs: Considerations of the DNA commission of the International Society for

Forensic Genetics (ISFG) on minimal nomenclature requirements. Forensic Sci Int Genet. 2016 May;22:54-63. doi: 10.1016/j.fsigen.2016.01.009.

12. Paz-García DA, Munguía-Vega A, Plomozo-Lugo T, Weaver AH. Characterization of 32 microsatellite loci for the Pacific red snapper, Lutjanus peru, through next generation sequencing. Mol Biol Rep. 2017 Apr;44(2):251-256. doi:10.1007/s11033-017-4105-4.

13. SantaLucia J Jr. Physical principles and visual-OMP software for optimal PCR design. Methods Mol Biol. 2007;402:3-34.

14. Schuelke M. An economic method for the fluorescent labeling of PCR fragments. Nat Biotechnol. 2000 Feb;18(2):233-4.

15. Vartia S, Collins PC, Cross TF, Fitzgerald RD, Gauthier DT, McGinnity P, et al. Multiplexing with three-primer PCR for rapid and economical microsatellite validation. Hereditas. 2014 Jun;151(2-3):43-54. doi:10.1111/hrd2.00044
